# Thermodynamic Fluid Equations-of-State

**DOI:** 10.3390/e20010022

**Published:** 2018-01-04

**Authors:** Leslie V. Woodcock

**Affiliations:** Department of Physics, University of Algarve, 8005-139 Faro, Portugal; lvwoodcock@ualg.pt

**Keywords:** equation-of-state, liquid-gas criticality, carbon dioxide, argon, water, SF_6_, virial coefficients

## Abstract

As experimental measurements of thermodynamic properties have improved in accuracy, to five or six figures, over the decades, cubic equations that are widely used for modern thermodynamic fluid property data banks require ever-increasing numbers of terms with more fitted parameters. Functional forms with continuity for Gibbs density surface ρ(p,T) which accommodate a critical-point singularity are fundamentally inappropriate in the vicinity of the critical temperature (T_c_) and pressure (p_c_) and in the supercritical density mid-range between gas- and liquid-like states. A mesophase, confined within percolation transition loci that bound the gas- and liquid-state by third-order discontinuities in derivatives of the Gibbs energy, has been identified. There is no critical-point singularity at T_c_ on Gibbs density surface and no continuity of gas and liquid. When appropriate functional forms are used for each state separately, we find that the mesophase pressure functions are linear. The negative and positive deviations, for both gas and liquid states, on either side of the mesophase, are accurately represented by three or four-term virial expansions. All gaseous states require only known virial coefficients, and physical constants belonging to the fluid, i.e., Boyle temperature (T_B_), critical temperature (T_c_), critical pressure (p_c_) and coexisting densities of gas (ρ_cG_) and liquid (ρ_cL_) along the critical isotherm. A notable finding for simple fluids is that for all gaseous states below T_B_, the contribution of the fourth virial term is negligible within experimental uncertainty. Use may be made of a symmetry between gas and liquid states in the state function rigidity (dp/dρ)_T_ to specify lower-order liquid-state coefficients. Preliminary results for selected isotherms and isochores are presented for the exemplary fluids, CO_2_, argon, water and SF_6_, with focus on the supercritical mesophase and critical region.

## 1. Introduction

There is a long history and an extensive literature of cubic equations-of-state, going back 140 years, from van der Waals renowned two-term equation for Andrew’s original p-V-T data on carbon dioxide [1], to current research and compilations with hundreds of terms and parameters. As the thermodynamic experimental measurements have improved in accuracy, ever more complex equations have evolved [2]. These equations are used to represent experimental thermodynamic properties for modern data banks such as the NIST compilation for CO_2_ [2,3]. Over recent decades, ever-increasing numbers of terms and more fitted parameters are required. The Span–Wagner equation for CO_2_ [2], for example, contains hundreds of terms, each with many fitted parameters, and various adjustable fractional exponents. As even more accurate data become available from future research, these functional forms will need yet more adjustable parameters.

**Figure 1:**
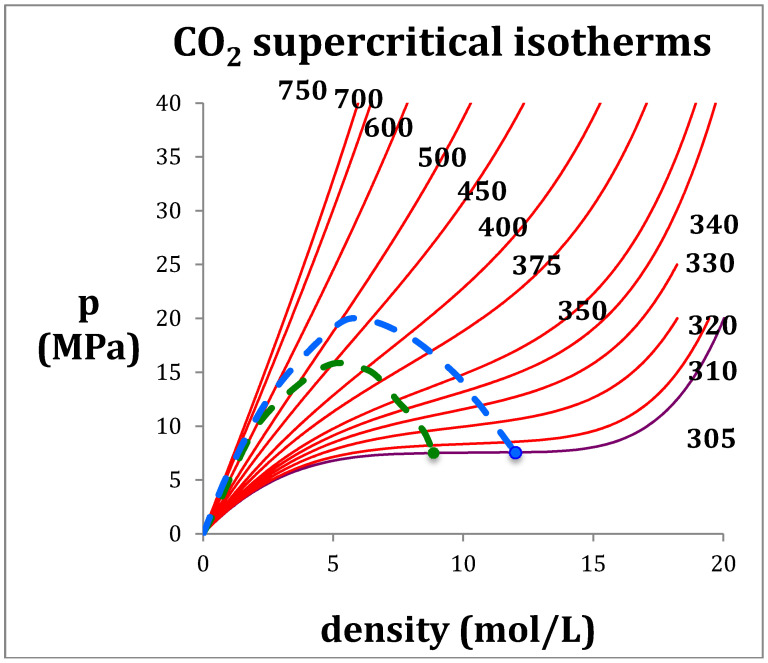
Supercritical T(K) isotherms for CO_2_: the green dashed line is the percolation loci PB; the blue dashed line is the percolation loci PA; the green and blue solid circles are the maximum experimentally observable coexisting gas density, and minimum coexisting liquid density along T_c_ i.e., 305 K (purple).

The basic reason of this inconvenient modeling practice is the van der Waals gas-liquid continuity hypothesis [1]. Continuous cubic functional forms are inappropriate in the vicinity of T_c_ and in the supercritical mid-range between gas and liquid phases. A mesophase, confined within percolation loci that bound the existence of gas and liquid phases by higher-order discontinuities, has been identified [4,5,6]. A simple numerical differentiation of NIST equations-of-state [7] can demonstrate the supercritical mesophase and observe the phase bounds, along any isotherm, of any fluid (e.g., CO_2_ Figure 1) for any of the 200 fluids in the NIST Thermophysical Property data bank. These boundaries have been smoothed over by the equations-of-state used to parameterize the original experimental data.

Within the uncertainties, p(ρ,T) is a linear function of ρ in the mesophase region. The origin of the linearity is the colloidal nature of the supercritical mesophase [6,8] with a linear combination rule for thermodynamic state functions, similar to subcritical lever rule for the inhomogeneous 2-phase region. When appropriate functional forms are used for gaseous and liquid states separately, we find that all other isothermal thermodynamic state functions in the mesophase are linear functions of density. In the case of pressure, the negative and positive adjacent deviations for liquid and gas on either side are quadratic. The parameterizations require only physical constants belonging to the specific fluid, Boyle temperature (T_B_), critical temperature (T_C_), and coexisting densities along the critical isotherm, and known virial coefficients b_2_(T) and b_3_(T) etc. A remarkable finding is that for the gas phases below T_B_, the coefficient b_4_ is effectively zero within the experimental uncertainty. There is rigidity symmetry between gas and liquid phases on either side of the mesophase reported previously [9], that relates the lower coefficients of the liquid equation to properties of the gas in the vicinity of the percolation loci.

The objective of this research is to investigate the extent that these findings can be used to describe experimental thermodynamic properties of a pure fluid over the whole range of equilibrium existence. Here, we investigate equations-of-state for gas, liquid and mesophase separately, each of which, in an initial approximation, require only measurable physical constants specific to particular fluids. Comparisons with experimental data are made via the NIST thermo-physical databank [3]. NIST tabulations reproduce experimental data with 100% accuracy, and therefore, provide a foremost criteria for testing the alternative description to van der Waals underlying science of critical and supercritical behavior of fluids as described in references [4,5,6,7,8,9].

## 2. Rigidity and Fluid-State Bounds

Rigidity, (ω)_T_, is the work required to isothermally and reversibly increase the density of a fluid. With dimensions of a molar energy, this simple state function relates directly to the change in Gibbs energy (G) with density at constant T

ω_T_ = (dp/dρ)_T_ = ρ(dG/dρ)_T_ (1)

The inequalities that distinguish gas from liquid are:

GAS   ρ < ρ_PB_     (d^2^ω/dρ)_T_ < 0 (2)

LIQUID   ρ > ρ_PA_    (d^2^ω/dρ)_T_ > 0 (3)

and for the mesophase

MESO   ρ_PB_ < ρ < ρ_PA_    (d^2^ω/dρ)_T_ = 0 (4)

**Figure 2:**
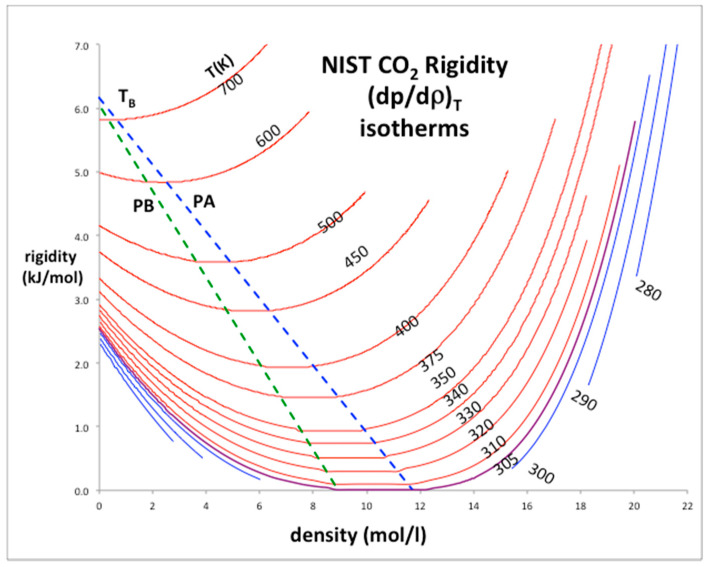
Rigidity of fluid phases of CO_2_ from NIST thermo-physical tables: the loci of gas and liquid-phase bounds are green (percolation line PB) and blue (percolation line PA) respectively; red lines are supercritical isotherms, blue lines are subcritical isotherms; the purple isotherm is T_c_ (305 K): the Boyle temperature (T_B_) = 725 K.

The rigidity isotherms shown in Figure 2 for CO_2_ have been obtained from NIST Thermophysical databank [3]. Inequalities in the derivatives of ω_T_ (Equations (2) and (3)) can thermodynamically define the percolation loci [9]. It follows that at low density the percolation loci must approach the Boyle temperature (T_B_) by its definition, which is obtained from the rigidity intersection interpolated at zero density. For CO_2_ the limiting rigidity (RT_B_: where R is the gas constant) along the percolation loci is 6.03 kJ/mol and T_B_ is 725 K, i.e., the temperature above which the 2nd-virial coefficient (b_2_) is positive and below which it is negative; at T_B_, p = ρkT and b_2_ = 0. In the pressure-density plane, the percolation loci exhibit maxima as density decreases and approach zero, i.e., the ideal gas limit at low pressures and densities. The same behavior is seen for other atomic (e.g., argon) and molecular (e.g., water) fluids.

It is clear from Equation (1) that ω ≥ 0, i.e., rigidity must always be positive although it can be zero in two-phase coexistence regions. Gibbs energy cannot decrease with pressure when T is constant. From these definitions moreover, not only can there be no “continuity” of gas and liquid but the gas and liquid states are fundamentally different in their thermodynamic description. Rigidity is determined by number density fluctuations at the molecular level, which have different but complementary statistical origins in each phase, hence the symmetry [9]. There is a distribution of many small clusters in a gas with one large void; there is a distribution of unoccupied pockets in the liquid with one large cluster. The statistical neighborhood properties of unoccupied pockets are the same as occupied sites.

**Figure 3:**
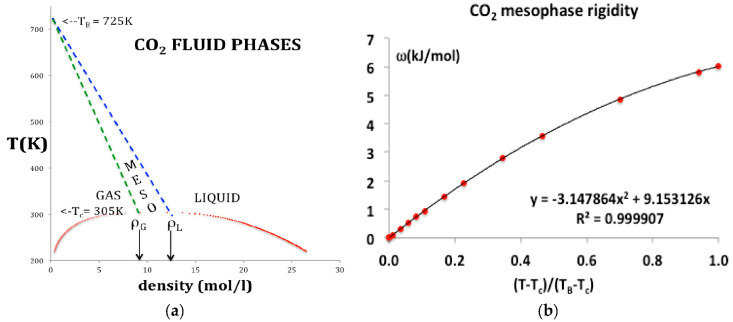
(**a**) Phase diagram for CO_2_: data obtained from NIST Thermophysical Properties compilation indicating the percolation loci that bound the supercritical liquid and gas states: the green and blue dashed lines are the gas (PB) and liquid (PA) percolation loci respectively [4,5,6,7,8,9]; solid red points; coexisting densities of the gas (ρ_G_) and of liquid (ρ_L_) at T_c_ are indicated by the arrows; (**b**) rigidity as a function of temperature in the mesophase.

The percolation loci and the critical coexisting densities at T_c_ for CO_2_ have been obtained by the method previously described in detail for Lennard-Jones fluids [5] and argon [8]. Looking at Figure 2, the coexisting densities of the gas (ρ_G_) and of liquid (ρ_L_) and the meso-density gap vary linearly between the critical temperature (T_c_) and Boyle temperature (T_B_). If the mesophase rigidity is obtainable in terms of T_B_ and T_C_, we can then represent the density bounds with linear functions and the rigidity in the mesophase as shown in Figure 3a,b, and incorporate a linear equation of state for the mesophase that connects with gas and liquid at the percolation bounds PB and PA respectively.

GAS    ρ_PB_(T) = ρ_G_(T_c_) [(T_B_ − T)/(T_B_ − T_C_)] (5)

LIQUID   ρ_PA_(T) = ρ_L_(T_c_) [(T_B_ − T)/(T_B_ − T_C_)] (6)

Use of original experimental data could result in non-linear Equations (5) and (6) for density state bounds in a more refined analysis. Given these expressions for the temperature dependence of the percolation loci, and the rigidity in the mesophase (Figure 3b), in terms of physical constants belonging to the specific fluid, we can now proceed to represent the thermodynamic surfaces using virial expansions.

The rigidity in the mesophase varies from kT at T_B_ to zero at T_c_. It is linearly dependent on temperature in the near-critical region up about 1.25 T/T_c_ but there is an increasing small quadratic term as seen in Figure 3b. The temperature dependence in the range T_c_ to T_B_ appears to be logarithmic, but without a scientific explanation, and within the uncertainty of the data, it is best represented by a simple quadratic

ω_T_ = c_1_T* + c_2_T*^2^ (7)

where T* = (T − T_c_)/(T_B_ − T_c_) and the constants c_1_ and c_2_ as given in Figure 3b.

## 3. Pressure Surfaces p(ρ,T)

The pressure for any thermodynamic equilibrium state point can be obtained as a function of density along any isotherm. The gas equation-of-state can be parameterized using the formally exact Mayer virial expansion [10]. The mesophase pressure increases linearly with density the region 0 > T > T_B_ and

ρ_PB_(T) > ρ < ρ_PA_(T), and for the ‘liquid’ state ρ > ρ_PA_(T), we can use an empirical expansion. The isothermal equations-of-state for pressure are as follows:

   GAS    p(ρ,T) = kTρ (1 + b_2_(T)ρ + b_3_(T)ρ^2^ + …) (8)

MESO    p(ρ,T) = p(ρ_PB_,T) + ω_T_ (ρ − ρ_PB_) (9)

LIQUID   p(ρ,T) = p(ρ_PA_,T) + [a_1_(ρ − ρ_PA_) + a_2_(ρ − _PA_)^2^ + a_3_(ρ − ρ _PA_)^3^ …] (10)

For the temperature-density region T_c_ < T < T_B_ and ρ < ρ_PA_(T), Equations (8)–(10) are based upon the observation that there is a phase transition of the third-order at the mesophase bounds with a discontinuity in the derivative of ω with respect to ρ, i.e., the third derivative of Gibbs energy with respect to ρ at constant T along an isotherm. For temperatures above T_B_, p(ρ_PA_,T) and ρ_PA_, in Equation (10), are zero, whereupon the coefficients a_n_ in Equation (10) become the same as the Mayer virial coefficients b_n_ (T) in Equation (8).

For the supercritical fluid at temperatures above T_B_, Equation (8) up to order b_4_ is sufficient to reproduce the pressure with five-figure accuracy, i.e., within the margin of the original experimental uncertainty, with a trend-line regression >0.999999, up to temperatures of 1000 K and pressures up to 50 Mpa. For the gas phase, at all temperatures below T_B_, the 4th virial coefficient is found to be essentially zero, within the uncertainty of the experimental data; all higher terms are negligible. Thus, for the case of CO_2_, the equation-of-state of the whole ‘gas’ region of Figure 3 requires just the second and a third virial coefficients b_2_(T) and b_3_(T) within the experimental precision.

An overall p(ρ,T) equation of state can be integrated along any isotherm to obtain the Helmoltz energy function A(T) and hence, via the partition function, all other thermodynamic state functions and/or derivatives by long-established procedures [11].

### 3.1. Supercritical CO_*2*_

In order to demonstrate a necessity of three separate equations-of-state, we take, as an example in the first instance, a supercritical isotherm T = 350 K, or T/T_c_ = 1.15, of CO_2_. The critical and Boyle temperatures for CO_2_ according to NIST databank [3] are T_c_ = 305 K and T_c_ = 725 K. Using the same method described previously for argon [8], the coexisting densities at T_c_ are found to be ρ_c_(gas) = 7.771 mol/L and ρ_c_(liq) = 13.46 mol/L. Substituting these physical constant values into equations (5 to 7) for T = 350 K we obtain the gas and liquid state density bounds ρ_PB_(gas) = 7.305 mol/L and ρ_PA_(liq) = 12.02 mol/L, and the rigidity ω(350 K) = 0.318 kT (0.925 kJ/mol).

If we try to fit the NIST data for the whole isotherm continuously from 0, just up to only 50 MPa, using a 6-term polynomial for example, we obtain the result:

p = −0.000008ρ^6^ + 0.000457ρ^5^ − 0.009373ρ^4^ + 0.096245ρ^3^ − 0.596317ρ^2^ + 3.459264ρ − 0.189239

with R² = 0.999125 

In this typical continuous overall polynomial fit, the lower order coefficients are scientifically meaningless. By contrast, by using virial coefficients and known properties of the mesophase bounds, the NIST data for the whole range of three regions can be reproduced with essentially 100% precision when the gas, liquid and meso-states have different equations-of-state. We assume that there is a third-order phase transition, i.e., a discontinuity in the third derivative of Gibbs energy at the gas and liquid-state boundaries in accord with inequalities (2 and 3).

**Figure 4:**
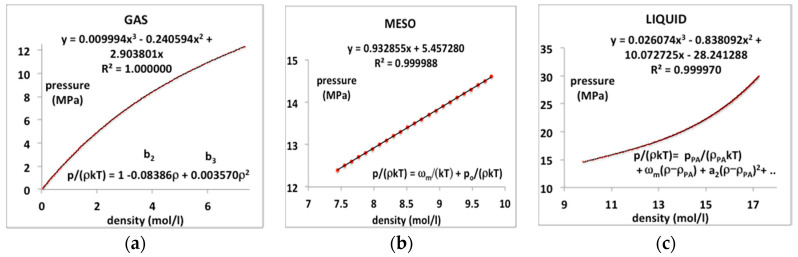
Pressure equations p(ρ) for CO_2_ at a supercritical temperature (350 K or T/T_c_ = 1.15) as derived from NIST Thermophysical Properties compilation: (**a**) gas state; (**b**) supercritical meso-phase and (**c**) liquid.

The experimental data from the NIST tabulations is reproduced with the same precision as the original data can be obtained, i.e., accurate to 5 figures, as evidenced by the mean-squared regression (R^2^) in the trendline polynomial coefficients as given. Also shown in Figure 4 are redefined equations to give the virial coefficients in Equations (8)–(10) as shown on the plots. The coefficient a_1_ in the liquid-state expansion is taken to be equal to the value of ω_T_ in the mesophase to effect the third-order discontinuity between the mesophase and the liquid state. There is a symmetry between the rigidity of gas and liquid on either side of the mesophase but at this stage, this observation is empirical. As it presently lacks formal theory, we assume only the linear coefficient in Equation (10) and make no assumptions regarding higher-order coefficients at this stage.

### 3.2. Supercritical Argon

Modern research into fundamental equation-of-state science is more often based upon the example of argon in the first instance because of the relative simplicity of the spherical two-body pair potential and the amenability to basic theory. The literature on equation-of-state data to high temperature and pressure is both extensive and accurate to at least five figures except in the vicinity of the critical temperature (T_c_) and pressure (p_c_) [3,12]. For temperatures above the Boyle temperature there appears to be just a single continuous fluid state up to the highest pressures measured, which, in the case of argon, is 1000 MPa, according to NIST [3,12]. If there are no discontinuities along isotherms for T > T_B_, the Mayer virial expansion is applicable over the whole accessible density range. The expansion for the pressure is

p = kT(ρ + b_2_ρ^2^ + b_3_ρ^3^ + b_4_ρ^4^ ……) (11)

and on differentiation the rigidity is

ω = (dp/dρ)_T_ = kT(1 + 2b_2_ρ + 3b_3_ρ^2^ + 4b_4_ρ^3^………) (12)

where b_n_ are the temperature-dependent virial coefficients with conventional definitions. The NIST data for four supercritical isotherms above and including the Boyle temperature (T_B_) can be precisely reproduced using Equation (12) and the virial coefficients determined. From Stewart and Jacobson [13] we obtain T_B_ = 414 ± 0.5 for argon, which is consistent with the zero value of the second virial coefficient obtained here from parameterizations of NIST rigidity values from sound velocity compilations.

**Figure 5:**
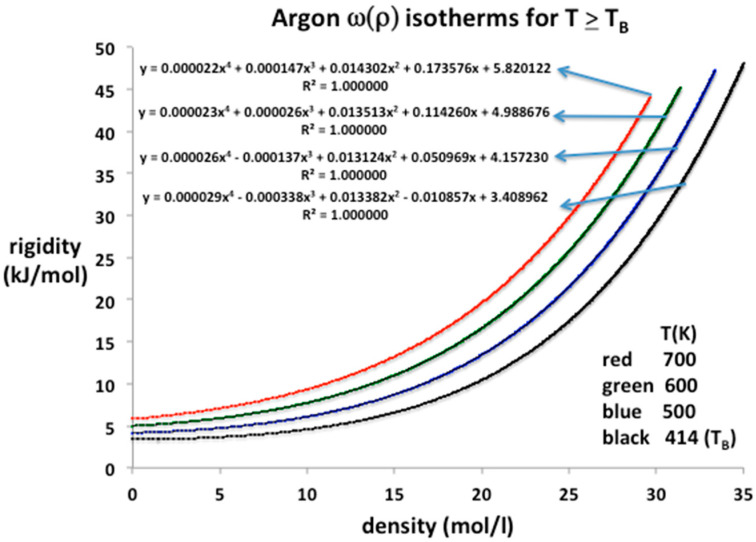
Rigidity isotherms, ω(ρ) for argon at temperatures from 700 K down to T_B_ (414 K) as obtained from NIST Thermophysical Properties compilation up to pressures of 500 MPa; all of the EXCEL trendline polynomials of order 4 reproduce the NIST isotherms with total precision, i.e., a regression of R^2^ = 1.000000 up to 6-figure precision.

The equation-of-state isotherms are presented in Figure 5. Also given are the polynomial trend-line parameters up to order 4. In this region, the entire experimental range of pressures, from near zero at ideal gas states at limiting low densities, up to 1000 MPa, can be represented to within the experimental five-figure precision by a three or at most four-term, closed-virial equation. Using Equation (12) we can deduce lower-order values of the virial coefficients of argon as listed in Table 1. The values obtained for b_2_ and b_3_ are in agreement with the literature values within the experimental uncertainties, which can be as high as 20% in the case of b_3_ [9]. For argon at supercritical temperatures, little is presently known experimentally about b_4_ and higher coefficients.

Lower-order virial coefficients, as defined by Equation (12), and calculated from the trend-line polynomial coefficients in Figure 5, are summarized in Table 1. All the experimental data from the NIST tabulations are reproduced with maximum precision as evidenced by the mean-squared regression in the trendline polynomial coefficients (R^2^ = 1.000000 to six decimal places in every case); the coefficients have been redefined to obtain values of the virial expansion coefficients defined in Equation (11). These are in line with previous b_2_(T) and b_3_(T) tabulations [14] within the uncertainties, which are rather wide, up to a few percent for b_2_(T), and up to 25% in the case of b_3_(T).

## 4. Critical Isotherms

### 4.1. Argon

**Figure 6:**
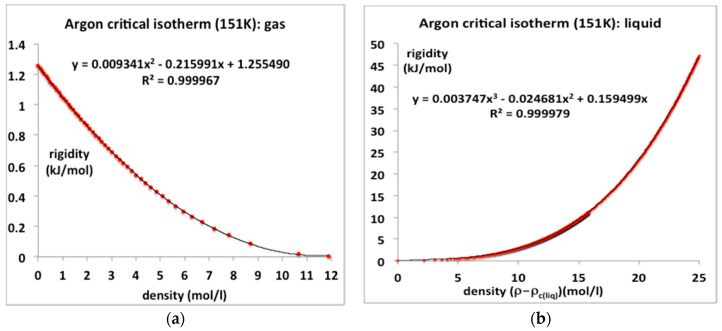
Rigidity isotherms, ω(ρ), for argon along T_c_ from NIST Thermophysical Properties compilation [3]: (**a**) gas phase from zero to maximum gas density at critical pressure 4.949 MPa (**b**) liquid phase from minimum liquid density from p_c_ (4.949 MPa) to 326 MPa. The rigidity along the critical divide is equal to zero.

The critical temperature of argon according to NIST is 150.87, the value obtained in a revised analysis of the data of Gilgen et al. is 151.1 ± 0.1. Here we represent the rigidities of the experimental 151 K isotherm from the NIST data tables using standard trend-line polymomials. The coexisting gas and liquid densities as obtained from the original experimental data of Gilgen et al. [14,15] are ρ_c_(gas) = 11.89 mol/L and ρ_c_(liq) = 14.95 mol/L [8]. For all temperatures between T_c_ and the triple point (T_tr_), the NIST ω(ρ)_T_ isotherms can be reproduced with 100% precision using just the two-parameter equation. Numerical values of the coefficients b_2_ and b_3_ at T_c_, obtained from the rigidity isotherms as illustrated in Figure 6a, are included in Table 1. The trend-line polynomials of order 3 reproduce the NIST isotherms over the whole range of equilibrium existence with high accuracy.

These trend-line equations for ω_T_(ρ) can be integrated to obtain pressure equations, and rewritten as:

Gas ρ < ρ_c_(gas) p = kT (ρ + b_2_ ρ^2^ + b_3_ ρ^3^) (13)

Meso ρ_c_(gas) < ρ < ρ_c_(liq) p = p_c_ (14)

Liquid ρ > ρ_c_(liq) p = p(ρ_cl_,T) + kT [a (ρ − ρ_cl_) + a_2_(ρ − ρ_cl_)^2^ + a_3_(ρ − ρ_cl_)^3^] (15)

where ρ_cl_ is the critical coexisting liquid density ρ_c_(liq). For argon, the critical pressure in Equation (14) p_c_ = 4.949 MPa [4]. Thus, we have an equation-of-state for argon along the whole critical isotherm, with no divergent singularity. The coefficients in Equations (13) and (15), are obtainable from the rigidity data plotted in Figure 6a,b, for example, by dividing the coefficients a_n_ by *n*kT.

### 4.2. Steam and Water

The evidence for supercritical percolation transitions bounding the existence of water and steam in the supercritical region, and the parameters describing the mesophase, have all been the subject of a previous article with an extended debate [16]. For the present purposes, we only need values of some physical constants that are given as an ancillary data list by NIST [3].

**Figure 7:**
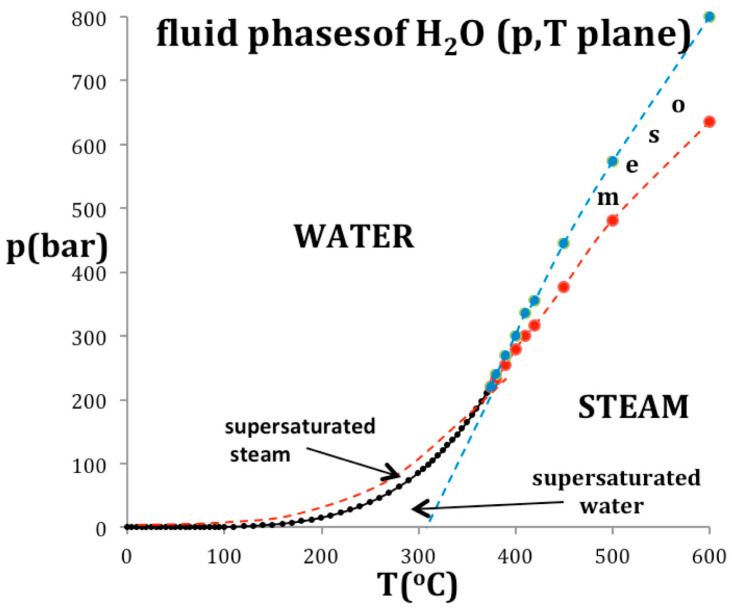
A phase diagram of water and steam in the p-T plane: the red and blue data points and dashed lines are the percolation loci state bounds PA (water) and PB (steam) respectively.

Figure 7 shows how the critical point in the T-p plane is thermodynamically defined by an intersection of two percolation loci, using the example of water. The coexisting density curves are taken from the experimental measurements of the IAPWS International Steam Tables [17]. The supercritical percolation transition points PA and PB intersect at T_c_, and continue to define the metastable limit of existence loci, usually referred to as spinodals, of the subcritical gas and liquid within the two-phase region. Values of the critical physical constants for water are: T_c_ = 647.1 K; p_c_ = 22.05 MPa; critical steam density ρ_cG_ = 13.02 mol/L; critical water density ρc_L_ = 20.61 mol/L [3,16].

Also plotted in Figure 7 are the experimentally observed spinodals [18] that bound the regions of metastable existence within the two-phase coexistence region at subcritical temperatures. At T_c_, the percolation loci, PA and PB in Figure 7, cross the critical coexistence line at its extremities to become the subcritical boundary of the metastable compressed gas (p > p_sat_) and metastable expanded liquid state (p < p_sat_) stability limits respectively. This behavior was also shown for liquid argon [8]. It is consistent with a phenomenological definition of PA and PB when both liquid and gas have the same values of the rigidity (ω_T_) on the same isotherm whereupon (d_2_p/dρ^2^)_T_ = 0 at both PA and PB but at different pressures. At these instability points, there is also zero surface tension and consequently no barrier to spontaneous nucleation of steam from water at PA, or water from steam at PB, for example.

**Figure 8:**
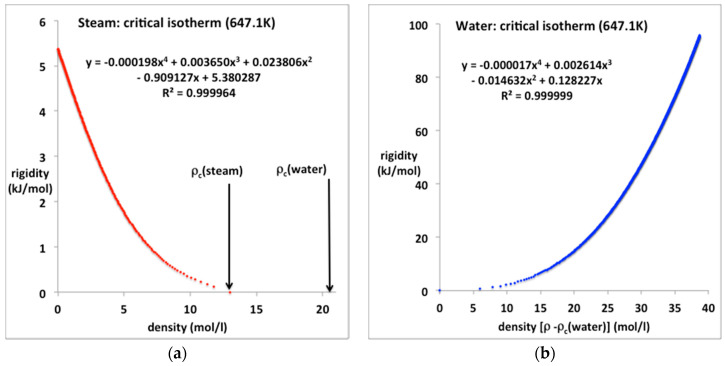
Rigidity (ω) for steam and water along the critical isotherm from NIST Thermophysical Properties compilation [4]: (**a**) steam from zero to ρ_cG_ at T_c_ (rigidity = 0); (**b**) liquid phase from minimum liquid density (ρ_cL_) at p_c_ = 22.05 MPa (ω = 0) to a pressure of 1000 MPa (ω ~ 100 kJ/mol).

The equations-of-state for steam and water along the critical isotherm are shown in Figure 8a,b respectively. In both cases a quartic equation is required to fit the data with the five-figure precision of the NIST tables. The fourth virial coefficient for steam at T_c_, however, still appears to be quite small in its contribution right up to the near critical density. We also note that the liquid water critical isotherm extends from a critical pressure of 22.05–1000 MPa and can be reproduced with six-figure precision by the quartic polynomial.

It is clear from the above that the equations-of-state for subcritical isotherms will require a scientific knowledge of the forms for the extension of the percolation loci into the subcritical two-phase region on the density surface. At present we do not have this information. It would be rather unwise to make any assumptions based only upon the known supercritical behavior of the density loci of PA and PB and rather scanty subcritical spinodals. In the following section, therefore, we will limit the present extension of the above to an analysis of the 100 K isotherm of liquid argon, to demonstrate the applicability of the simple equation-of-state forms Equations (8)–(10) to subcritical gas and liquid state isotherms.

## 5. Subcritical Isotherms

### Subcritical Argon

**Figure 9:**
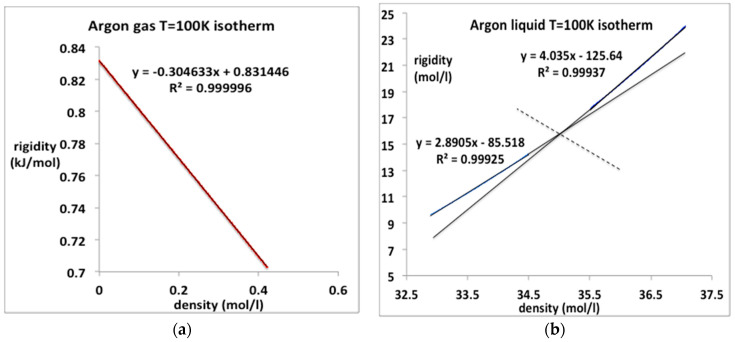
Rigidity ω(ρ) for argon along the sub-critical isotherm T = 100 K from NIST [4]: (**a**) gas phase from zero to maximum equilibrium gas coexistence density (0.42 mol/L): (**b**) liquid phase from minimum liquid equilibrium coexistence density (32.9 mol/L) at the coexistence pressure (MPa) to a pressure of 100 MPa.

The 100 K (T/T_c_ ~ 2/3) isotherm data for the rigidity from the NIST compilation of gaseous and liquid argon are re-plotted in Figure 9. The gas pressure up to the condensation line can be represented with just the two-term equation with the value of b_2_ given in Table 1; b_3_ is essentially zero. Thus the accurate equation for argon gas at lower temperatures is simply

p_gas_ (100 K) = kTρ (1 + b_2_ ρ) (16)

The value obtained from the linear fit of ω_T_ in Figure 8a, is 183.2 × 10^−3^ mol/L compares favorably with an experimental value −187 (cm^3^/mol) obtained in 1958 [13].

The corresponding pressure data for liquid argon at 100 K up to freezing (62 MPa) may not be continuous in all its derivatives, and hence the equation-of-state may not be as simple as it looks if there is a higher-order discontinuity. The liquid isotherm appears to comprise two linear parts containing an intersection at an intermediate density of around 35.0 mol/L. This is consistent with a previous conjecture [19] that as the crystallization point is approached, the liquid itself may become another kind of mesophase as large clusters of ordered arrangements of atoms, probably bcc in the case of argon, begin to stabilize. This could be a feature of the equilibrium “liquid” state structure in the near triple-point density region. One can regard the data in Figure 9b as a fragment of evidence for this conjecture. We will not consider this intriguing possibility further at this stage. We note, however, that if this is indeed the underlying science, any equation-of-state for the equilibrium ‘pure liquid’ may terminate before the equilibrium crystallization density is reached along subcritical isotherm, below around 115 K in the case of argon.

## 6. Energy and Heat Capacities

### 6.1. Argon Critical Isotherm and Isochores

The only real evidence for a van der Waals-like singularity and a critical point, with continuity of gas and liquid [1] and universal scaling properties [20], is evidently in historic heat capacity measurements [21,22]. Here we focus briefly at the equation-of-state for the internal energy U(ρ)_T_ for the critical isotherm of argon close to T_c_ (151 K). The NIST data with the trend-line parameters are shown in Figure 9. The foremost observation is that along this isotherm the gas phase is represented by a quadratic requiring two coefficients whereas the liquid phase is linear up to a density around 35 mol/L. The critical divide is linear by definition in accord with the lever rule or proportionality.

**Figure 10:**
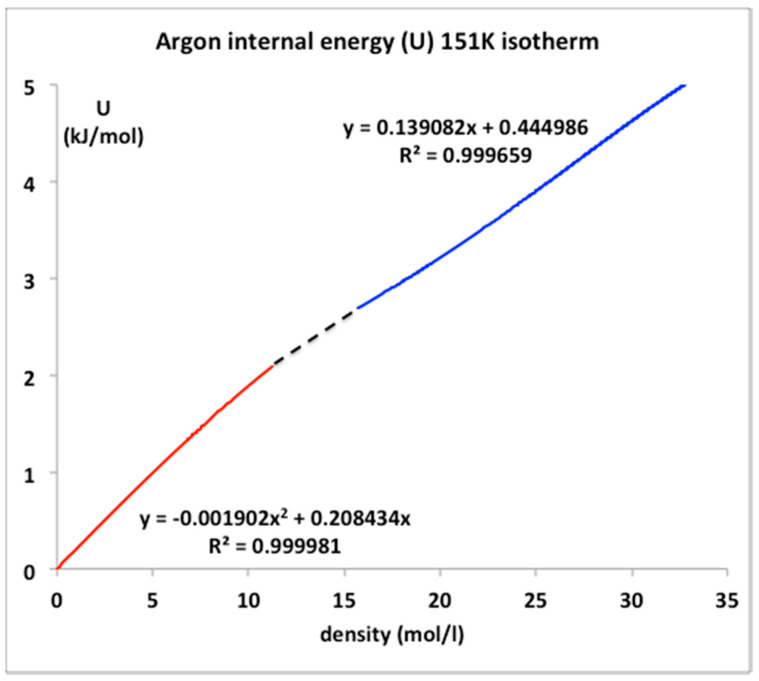
Internal energy U(ρ) for argon along the isotherm (T = 151 K) using data from NIST [4].

**Figure 11:**
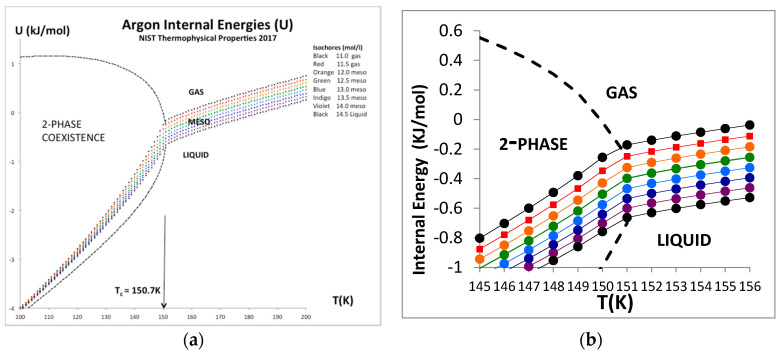
(**a**) Internal energy isochores, U(T)ρ for argon for a temperature range from near triple point to supercritical from NIST [4] showing an expanded window picture of the near critical isochores to the right. (**b**) Magnified data in critical region showing a weak discontinuity in (dU/dT)_v_, i.e., Cv, at T_c_ but clearly no evidence of logarithmic divergence on either side of T_c_.

The internal energy for all temperatures below T_c_ in the two-phase region is defined to be linear by the lever rule since the physical state is of two-phase coexistence. Above the critical divide the three regions shown in Figure 10, however, look much the same, so we can identify two higher-order phase transitions bounding the mesophase. There is no evidence of divergent behavior of slope of U(T)ρ, i.e., in the isochoric heat capacity C_v_. Isotherms from T_c_ to 2T_c_ all show a constant value of (dU/dT)ρ in the supercritical region, as seen in Figure 11. Along the critical divide, the isochoric heat capacity is defined by the lever rule: for T ≤ T_c_

C_v_ = (dU/dT)_V_ = X C_v_(liq) + (1 − X) C_v_(gas) (17)

where X is mole fraction of liquid.

Uhlenbeck [20] appears to have been the first to recognize what he described as “surprising Russian results” [21,22] as being supportive of a universal picture of critical phenomena in which the isochoric heat capacity C_v_ is believed to diverge logarithmically on both sides of the critical point. When the proceedings of the Washington Conference were published one-year after the event [20], the articles by Rowlinson, and by Fisher, in particular, hailed the Russian discovery as a breakthrough (referenced by Rowlinson though curiously not referenced by Fisher), with the implicit proclamation that the van der Waals critical point, with continuity of liquid and gas, had become established scientific truth, and universal scaling properties similar to a 2D-Ising model lattice gas would describe gas-liquid and indeed all similar ‘critical point singularities’. In fact, an abundance of experimental evidence against this conjecture was already there in the literature [23] and not supportive of van der Waals hypothesis or universality.

Despite using complex equations-of-state that could accommodate the concept of a critical density at the node of a parabola, the NIST thermodynamic data bank is still valid as “experimental” evidence that the 1965 Washington proclamation of ‘a universality theory’ was fundamentally incorrect. It was based upon a misinterpretation of the isochoric heat capacity C_v_ for T < T_c_, and spurious results at, or millikelvins greater than, T_c_ [24,25]. Along any isochore within the critical divide there can be no divergence of the isochoric heat capacity. For T > T_c_ a divergence of C_v_ would be a contradiction of laws of thermodynamics. If one could reversibly add heat (Q_rev_) to a classical Gibbs fluid system that does no work with no increase in the temperature, it would be imply, for example, that Q_rev_ i.e., enthalpy, or (Q_rev_/T) i.e., entropy, are not state functions [26].

### 6.2. SF_6_ near Critical Isochors

**Figure 12:**
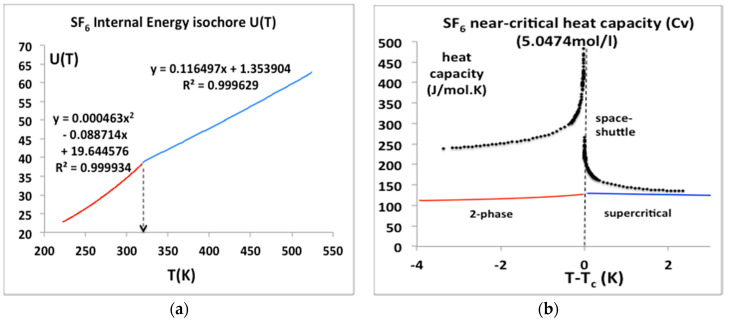
(**a**) Internal energy isochores, U(T)ρ for SF6 for a temperature range from near triple point to supercritical from NIST Thermophysical Properties compilation [4]; (**b**) Isochoric heat capacities calculated from the equations in Figure 1a, i.e., (dU/dT)ρ alongside the original space shuttle heat capacity data points.

Sulphur hexafluoride has a special importance in this scientific debate; its critical temperature and pressure are amenable to experiments at near ambient conditions. It has been the subject of a microgravity experiment aboard the NASA Space Shuttle [27] that appeared to confirm the erroneous result obtained some 25 years earlier by Voronel and his colleagues [24,25]. We have obtained the original published data points of the space shuttle experiments (see acknowledgements) and have plotted them alongside the U(T)_V_ equation-of-state from the NIST data bank parameterized as a simple quadratic for T < T_c_ and linear for T > T_c_ equations in Figure 12a. The equations for U(T) at the same density as the space shuttle μg experiment can be differentiated to give the isochoric heat capacities C_v_ in Figure 12b. In contrast to both the historic Russian results for argon, and the space shuttle “C_v_” values also plotted in Figure 12b, there is no divergence of the isochoric heat capacity _Cv_ either below or above T_c_, as suggested, for example, in alternative near critical equations of state with a crossover to the divergent form in the vicinity of T_c_ [28].

We can see from Figure 12 that the isochoric heat capacities reported in the Russian experiments, and the space shuttle experiments, as relied upon by all the principle proponents of universality for several decades [20], are a misinterpretation of the correct thermodynamic definition of the isochoric heat capacity C_v_ below the critical temperature. If the equations-of-state for U(T) in Figure 12a are essentially correct, the space shuttle result, as well as the argon results for “C_v_” must be wrong in the region above T_c_. One explanation may be that the experimental measurements on argon [21] were carried out on continuously stirred steady-states. Heat capacity data obtained for stirred samples in closed adiabatic calorimeters cannot be construed as equilibrium thermodynamic data for the determination of fluid equations-of-state in the close proximity of T_c_, either below or above T_c_, for any isochore along the critical divide. In the case of the SF_6_ microgravity experiments [22], if there was no stirring, there must be some other reason for the discrepancy seen in Figure 12, that is presently inexplicable.

## 7. Conclusions

From the foregoing analysis, we conclude that the pressure equation-of-state, and hence all other thermodynamic state functions, can be expressed simply in terms of a few physical constants belonging to the fluid, and coefficients in virial expansions. The van der Waals equation-of-state survived for about 50 years until, increasingly, there was evidence that it could not account for the experimental data unless it was extended to include more and more terms and adjustable parameters. Modern equations-of-state with a large number of terms and adjustable parameters can reproduce experimental p-V-T data to high precision for chemical engineering applications, but they may no longer reflect an underlying physical science in the spirit of van der Waals.

We have found that the equation-of-state for the low density gas phases, in all three temperature regions T > T_B_, T_B_ >T > Tc, and T < T_c_, can all be represented by the first two or three terms of a Mayer virial expansion. For T > T_B_ a single virial equation can represent the whole isotherms up to the highest recorded pressures, which in the case of argon is 100 MPa. For supercritical temperatures below the Boyle temperature the gas phase can be represented by a term virial expansion to within the accuracy that the original pressures reported by NIST [3]. The virial equations-of-state are fundamental to all atomic and molecular fluids and appear to be accurate up to second order, i.e., to quadratic terms for all temperatures below T_B_. We will report detailed comparisons and virial coefficients for the case CO_2_, and also some preliminary comparisons with experimental data and NIST equations, for the exemplary fluid argon, in due course.

At this stage in the development of science-based alternative forms of the equation-of-state we have not attempted to parameterise the second and third virial coefficients as functions of temperature. We note, however, there is evidence that b_2_(T) may be non-analytic at the Boyle temperature. It can be represented by two different series expansions, at least in the case of a simple Lennard–Jones model, above and below T_B_ [29,30]. There are also some indications that b_3_(T) exhibits a maximum at or near T_c_. Also, we find that for simple fluids, e.g., argon, below T_B_, the coefficient b_4_(T) appears to become vanishingly small. A more accurate determination of lower-order virial coefficients from original thermodynamic data ought now to become a research priority.

## Figures and Tables

**Table 1:** Argon virial coefficients.

T (K)	b_2_ (10^−3^ mol/L)	b_3_ (10^−6^ mol^2^/L^2^)	b_4_ (10^−9^ mol^3^/L^3^)
700	14.91	0.8191	0.0063
600	11.45	0.9029	0.0013
500	6.130	1.0523	−0.0082
414 (~T_B_)	0.0081	1.3085	−0.0248
151 (~T_c_)	−86.02	2.48	0
100	−183.2	0	
